# Interactive robot teaching based on finger trajectory using multimodal RGB-D-T-data

**DOI:** 10.3389/frobt.2023.1120357

**Published:** 2023-03-16

**Authors:** Yan Zhang, Richard Fütterer, Gunther Notni

**Affiliations:** ^1^ Group for Quality Assurance and Industrial Image Processing, Technische Universität Ilmenau, Ilmenau, Germany; ^2^ Fraunhofer Institute for Applied Optics and Precision Engineering IOF Jena, Jena, Germany

**Keywords:** multimodal image processing, RGB-D-T-data, point cloud processing, finger trajectory recognition, robot teaching, meshless finite difference solution

## Abstract

The concept of Industry 4.0 brings the change of industry manufacturing patterns that become more efficient and more flexible. In response to this tendency, an efficient robot teaching approach without complex programming has become a popular research direction. Therefore, we propose an interactive finger-touch based robot teaching schema using a multimodal 3D image (color (RGB), thermal (T) and point cloud (3D)) processing. Here, the resulting heat trace touching the object surface will be analyzed on multimodal data, in order to precisely identify the true hand/object contact points. These identified contact points are used to calculate the robot path directly. To optimize the identification of the contact points we propose a calculation scheme using a number of anchor points which are first predicted by hand/object point cloud segmentation. Subsequently a probability density function is defined to calculate the prior probability distribution of true finger trace. The temperature in the neighborhood of each anchor point is then dynamically analyzed to calculate the likelihood. Experiments show that the trajectories estimated by our multimodal method have significantly better accuracy and smoothness than only by analyzing point cloud and static temperature distribution.

## 1 Introduction

Nowadays, robots are already capable of supporting humans for some precise or dangerous tasks in a wide variety of fields, such as assembly robots, welding robots and medical robots. In general, robots need some customization and system integration to satisfy such specialized tasks, which requires users to have some expertise in robot operating. In this respect, the most common basis task is trajectory teaching. In order to respond to evolving industrialization levels, a modern dynamic production line requires an efficient approach for robot trajectory generation. For this purpose, robot developers and manufacturers have been trying to work on teach pendant. However, by using this device, the teaching of a complex arbitrary trajectory containing an extremely high number of waypoints is very time-consuming. If it needs to be more efficiently solved, a professional programmer is required. Therefore, an easy-to-use and still effective trajectory teaching approach becomes a research hotspot in recent years to lower the employment barrier for such skill based professions.

Regarding this application, there have been many studies in recent years. For example, in the research (Braeuer-Burchardt et al. (2020)), a demonstrator system for selected quality checks of industrial work pieces with a human machine interaction was proposed, in which the check position of a work piece is determined by a finger pointer. In addition, the company Wandelbots Teaching (Wandelbots (2022)) developed a robot teaching system using a TracePen as an input device. The pen works like a tracker for recording a sequence of waypoints (rotations and translations under the robot base coordinate system) that can be further refined manually by their software.

In this article, we will propose a vision-based trajectory teaching method. In our approach, the core module is a finger trajectory recognizer, which is realized by using a multimodal vision sensor system. It consists1 of a color camera (RGB), a 3D sensor (D) and a thermal camera (T) for multimodal point cloud (RGB-D-T) recording. The touch of an object with a finger results in a slight temperature change of the object surface. If the finger is moved on the 3D-object along the robot’s imaginary motion path it leaves a heat trace on the object surface resulting in a 3D-heat-trajectory, which directly represents the robot motion trajectory.

To get an accurate 3D-trajectory one has to take into account that mostly finger trajectory recognizers are based on hand detection or human skeleton detection, for example, (Halim et al. (2022); Du et al. (2018)). Such approaches are usually inaccurate because at the moment when the finger and the object are in touch, the actual contact point will definitely be blocked by the finger at the camera’s perspective. In our method, introducing multimodal point cloud analysis, the finger movement process can be considered as a heat transfer process caused by a moving Gaussian point heat source. By analyzing the residual heat on the object surface, the trajectory can be predicted more accurately. In recent years an increasing number of multimodal sensor-based image processing methods have been discussed and applied to scenarios with human interaction. For example (Jeon et al. (2016)), introduced an outdoor intelligent surveillance system with a color and a thermal camera, which is capable of recognizing humans in both day and night. In most approaches, temperature is analyzed as a static feature in the same way as color. In fact, in comparison to color, even if the heat source is fixed or removed, temperature still changes relative to time and spatial variables. More attention should be paid to these characteristics in order to extract more information from multimodal point cloud to achieve more diverse human-machine interactions. Therefore, according to the heat equation, a node in a temperature field is in a heat dissipation state when it has a negative divergence. The greater its absolute value, the higher the rate of heat transfer. Thus in our method the node with a low divergence will be considered as a candidate of contact point with a high probability.

In this regard, the temperature analysis in 2D thermal images is limited. By using a 2D camera, an temperature field can only be accurately captured when the surface of the object is a plane and parallel to the sensor plane. Otherwise, the spatial independent variables (x-, y- and z-coordinates in the world coordinate system) used to calculate divergence will be non-uniformly observed by a 2D camera. This unevenness is related to the complexity of the object surface and the placement posture of the object. It leads to errors in the solution of gradient or divergence. By using 3D point cloud analysis, this problem can be avoided. However, a point cloud is a meshless and unordered point set. Common finite difference methods such as the central difference formula cannot be used directly to calculate the numerical solution of the partial differential. Therefore, in this paper we propose a fast method to find the approximated solution of the divergence for each node in a meshless 3D temperature field (a thermal point cloud).

In overall terms, our approach follows Bayesian theory. A candidate region (prior probability) is firstly determined with the help of hand/object semantic segmentation in multimodal point cloud. Then a distribution of the divergence (likelihood) is calculated in candidate regions. Finally, the by finger obscured contact points (posterior probability) will be estimated. By using these contact points, a realistic robot motion trajectory is generated through interpolation. In the experimental section, the error of the approximated divergence solution and the deviation of robot motion trajectory generation will be evaluated and discussed.

## 2 Related work

Currently, most industrial robot manufacturers provide a teach pendant with a manual motion mode, with which the robot can be moved manually to a set of positions they are marked as waypoints. The robot can then be simply programmed to execute a trajectory consisting of these waypoints in sequence. As mentioned above, it will be particularly time consuming when complex and arbitrary trajectories with a large number of waypoints are defined. In this regard, teaching by human body language has become a popular area of research. The previous works (Braeuer-Burchardt et al. (2020)) and (Wandelbots (2022)) demonstrate the application of modern human-machine interaction methods for straightforward robot teaching. However, they have some limitations. (Braeuer-Burchardt et al. (2020)) brings a contactless interaction, but the exact check position cannot be obtained by only a finger pointer. The method of (Wandelbots (2022)) can avoid this problem, but an expensive TracePen is required to achieve the tracking. Other than that, in the works of (Liu et al. (2022); Jen et al. (2008); Yap et al. (2014); Manou et al. (2019); Pratticò and Lamberti (2021)), Virtual Reality (VR) technology was used to improve the human-robot interface without the requirement for complicated command or programming. Such as in the studies (Su et al. (2018); Abbas et al. (2012); Stadler et al. (2016); Pettersen et al. (2003)), Augmented Reality (AR) systems were designed to allow users to govern the movement of real robots in a 3D space *via* a virtual one generated through AR technology. Whether in the application using VR or AR, the recognition of finger trace always plays the role of a bridge between realistic movements and virtual trajectories. The most intuitive solution to solve this core task is hand skeleton recognition based on color or depth image, such as (Halim et al. (2022); Baek et al. (2018); Cheng et al. (2021); Zhang et al. (2020); Osokin (2018)). However, the finger trace defined by these methods is not exactly equivalent to the robot motion trajectory on the object surface. Due to the occlusion by finger, the trajectory cannot be captured in real time by cameras. Hence, we recommend introducing multimodal sensors (RGB-D-T) to collect more diverse information, in order to improve prediction results closer to true trajectories.

Guanglong et al. (Du et al. (2018)) introduced a particle filter and neural network based gesture estimator using a multimodal sensor system containing a RGB-D camera and an inertial measurement unit, in which multimodal information including RGB-D image, velocity and acceleration of hand as well as speech are fused. Then a neural network is used to encode such data to predict the finger trace. In the research of Zhang et al. (Zhang et al. (2021)), by using PointNet (Qi et al. (2017a)), PointNet++ (Qi et al. (2017b)) and RandLANet (Hu et al. (2020)), multimodal image data with color, thermal and point cloud was encoded and decoded to perform a pixel-wise hand/object semantic segmentation for application of a hand over robot. Their experimental results showed a better segmentation performance of the hand-object interaction region with the help of thermal information compared to RGB-D-based segmentation, if the object has a similar color or temperature as the hand especially. However, temperature is analyzed only as a static feature, like color. In other words it should be considered as a multispectral 3D image analysis.

## 3 System overview

**FIGURE 1:**
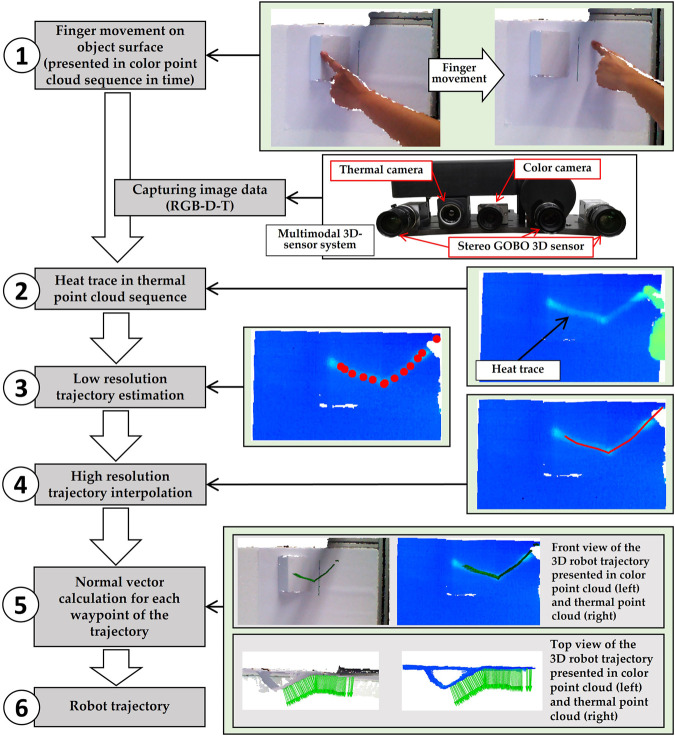
Basic concept of our approach.

Figure 1 shows the basic concept of our approach. At first (step 1) the finger touches the object and moves along an imaginary robot trajectory to teach in. During this movement the multimodal 3D-Sensor consisting of a 3D-sensor, a RGB-camera and a thermal camera (picture right) capturing a series of RGB-D-T data resulting in a 3D-heat trace data set (step 2). With the help of these collected information, the trajectory with low resolution is estimated (step 3) and then used to interpolate a dense smooth 3D motion trajectory for robots (step 4). By using the 3D-data of the object, the orientation of each waypoint of the 3D-trajectory will be recalculated, which is equal to the surface normal vector at its position, to ensure that the robot can always move perpendicularly to the object surface (step 5). Finally, this high-resolution trajectory is used as the input for an identical movement of the robot (step 6).

**FIGURE 2:**
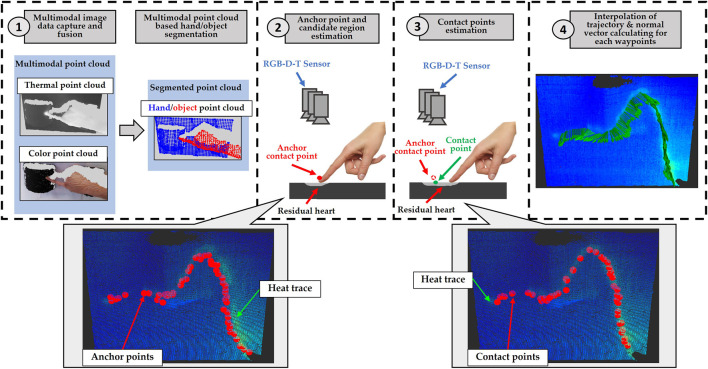
Method architecture.

As mentioned above, the core component of our approach is a finger recognizer. A finger trajectory recognition can be regarded as a branch of the task of object (contact point) tracking. The process of such tasks is often described as a hidden discrete-time Markov chain and a number of solutions for this are theoretically based on Bayesian theory. The concept of such solutions is divided into three steps. Firstly, the prior probability is inferred from the system model. Then the likelihood is estimated based on the observation model. Finally, the posterior probability is calculated dependently on the prior probability and the likelihood, which will be used to produce the prediction for the target random event. Our system is also developed along this lines, in which a random event of whether a point in a candidate region is a contact point will be predicted. As shown in the Figure 2, our finger recognizer schema is divided into four modules. In module 1, see chapter 4, the multimodal sensor system and robot base need to be calibrated in order to calculate intrinsic parameters and extrinsic parameters of cameras and the robot. They will be utilized to fuse multimodal 3D image data as well as to interconvert 3D waypoints (orientation and position) between the camera and robot coordinate systems. Then the hand and objects will be semantically segmented in point cloud. In module 2, see chapter 5, by using the segmentation results, a set of anchor finger-object contact points on time series is estimated roughly, and a neighborhood searching is performed for each anchor point to determine a local candidate region. The prior probability *P*_*prior*_ is calculated for each point in the candidate region. Furthermore, based on these local candidate regions, the computational complexity of estimating the real contact points is reduced significantly. Module 3 (see chapter 6) provides thermodynamic analysis in each candidate region to obtain the likelihood distribution *L*. It is worth mentioning here that we introduce a divergence-based temperature field analysis, which has a noticeable advantage over the analysis based only on temperature distribution for trajectory estimation. Meanwhile it is verified in the experimental chapter 8. Finally, the true contact points are predicted by calculating posterior probability

$$P_{\mathrm{posterior}}=w_{1}P_{\mathrm{prior}}+w_{2}L,$$ (1)

where *w*_1_ and *w*_2_ are the weights for prior probability and likelihood. In our experience, setting these weights needs to consider the heat transfer coefficient of the object and the speed of finger movement. Finally, the finger-object contact points will be defined as

$$ContactPoint=argmax\left(P_{\mathrm{posterior}}\right).$$ (2)

In module 4, see chapter 7, a high-resolution trajectory will be interpolated based on these contact points in order to achieve smooth robot motion.

## 4 Multi-modal point cloud fusion and segmentation (module 1)

### 4.1 Multimodal sensor system

As shown in Figure 1, our multi-modal 3D imaging system consists of a high-resolution active stereo-vision 3D sensor based on GOBO (Goes Before Optics) projection (Heist et al. (2018)), a color camera (Genie Nano C1280 (Genie (2022))) and a thermal camera (FLIR A35 (FLIR (2022))).

### 4.2 Multimodal image data fusion and hand object segmentation

Inspired by (Zhang et al. (2021)), the multimodal sensor system was calibrated using a copper-plastic chessboard as the calibration target. The multimodal image data was then fused using the intrinsic and extrinsic parameters as well as further hand-object segmented using RandLANet (Hu et al. (2020)). RandLANet is a lightweight neural network with a multi-level architecture designed for large-scale 3D point cloud semantic segmentation. In each level, a random downsampling is used to enable that the point density of the point clouds is progressively decreased. By using a local spatial encoding module (LocSE) in each neighborhood, XYZ-coordinates of all points, Euclidean distances as well as XYZ-differences between the centroid point and all neighboring points are explicitly encoded using shared multi-layer perceptron. Additionally, in between two adjacent levels, an attentive pooling is utilized to aggregate the features. Then, multiple LocSE and attentive pooling units with a skip connection are stacked as a dilated residual block, which is repeatedly used in the RandLANet. A hierarchical propagation strategy with distance-based interpolation and a cross level skip links is adopted to upsample the point clouds to the original size.

The experimental results in (Zhang et al. (2021)) indicate that based on an XYZ-RGB-T (XYZ: spatial coordinate, RGB: color, T: thermal) point cloud, the RandLANet can learn the complex aggregation and combination of multimodal features. In this way, the information from each channel compensates for their respective weaknesses. The segmentation of the XYZ-RGB-T point cloud has better robustness than the XYZ-RGB and XYZ-T for some objects that have similar color, surface texture, or temperature as the hand. For example, the heat trace left on the object by the finger did not worsen the segmentation results. This statement is an important premise for the application of this article.

## 5 Calculating the candidate region (module 2)

### 5.1 Anchor points estimation

**FIGURE 3:**
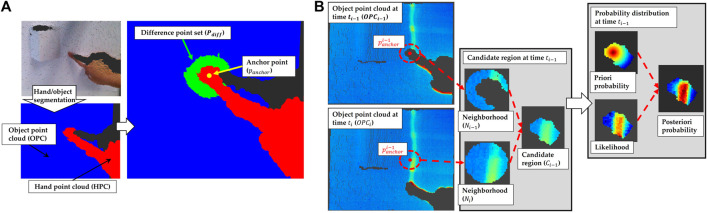
**(A)**: hand/object segmentation and the method for defining anchor points (*P*_*anchor*_) **(B)**: the concept to define the candidate region (*C*_*i*_) and a visualization of the prior probability (*P*_*prior*_), likelihood (*L*) and posterior probability (*P*_*posterior*_).

By using the segmented hand and object point clouds (*HPC* and *OPC*), a number of anchor points can be simply determined by the mean of the nearest point pair between them. However, such an anchor point is not optimal, because it will be identified as a point on top of the fingertip rather than a point on the object surface (contact area between finger and object). On the other hand, the 3D points within these areas usually cannot be reconstructed by a 3D sensor due to occlusion. Hence, in order to estimate an anchor point $p_{\mathrm{anchor}}^{i}$ that is closer to the true contact point at time *t*_*i*_, we use two different distance thresholds *d*_1_ and *d*_2_ to segment two point clouds *P*_1_ and *P*_2_ from the object point cloud *OPC* ^*i*^. They consist of a number of object points whose distance to their nearest hand points is less than *d*_1_ and *d*_2_. Since both *P*_1_ and *P*_2_ are subsets of *OPC* ^*i*^, then a difference set *P*_*diff*_ = *P*_2_\ *P*_1_ can be calculated using set operator simply. *P*_*diff*_ is approximately an annular point cloud whose centroid will be defined as the anchor point $p_{\mathrm{anchor}}^{i}$ , as shown in Figure 3A. Details of the algorithm will be given in Supplementary Appendix SA.

### 5.2 Candidate regions estimation

Obviously, at time *t*_*i*_, the candidate region *C*_*i*_ should be located in the spherical neighborhood *N*_*i*_ of the anchor point $p_{\mathrm{anchor}}^{i}$ . As shown in the Figure 3B, at the time *t*_*i*−1_ in *N*_*i*−1_, there is an area that was obscured by the finger. This area will be observed at the time *t*_*i*_ in *N*_*i*_. It will be defined as the candidate region *C*_*i*−1_. For calculating *C*_*i*−1_, we need to determine the difference set of *N*_*i*−1_ and *N*_*i*_. However, *N*_*i*−1_ and *N*_*i*_ were captured at different times, thus this difference set cannot be calculated by using set operators. We introduce a tolerance *r*_*c*_. If a point in *N*_*i*_ has a nearest point in *N*_*i*−1_ and their distance is less than *r*_*c*_, this point will be considered that it has an approximate overlapping point in *N*_*i*−1_. Then a point cloud consisting of a number of points in *N*_*i*_ without overlapping points in *N*_*i*−1_ will be defined as the candidate region *C*_*i*−1_ at time *t*_*i*−1_. In other words, the range of *C*_*i*−1_ is determined at time *t*_*i*−1_, while the information (point position and temperature) is acquired at time *t*_*i*_. The details of this algorithm will be explained in Supplementary Appendix SB.

### 5.3 Prior probability calculation

In each candidate region, a prior probability density function related to the distribution centered on the corresponding anchor point can be defined as

$$P_{\mathrm{prior}}\left(x\right)=\int G\left(x\right)C\left(x\right)dx,$$ (3)

where *x* denotes the position of random variables in a domain. In our case that is the 3D coordinates of the object points in the candidate region. *G*(*x*) denotes a Gaussian probability density function and *C*(*x*) denotes a function to describe the relationship between the distribution of the candidate points and the probability whether they are true contact points. In which, a point has the probability that is negatively correlated with its distance to the anchor point. In other words, a point closer to the anchor point has a higher probability, as shown in Figure 3B.

## 6 Calculating the optimized contact point (module 3)

In this section we discuss how to solve the divergence of each point in the candidate region. Based on the divergence, the likelihood is further calculated to complete the Bayesian approach. In this regard, the candidate region can be considered as a local temperature field, in which the finger can be considered as a moving Gaussian point heat source. The heat transfer state of the residual heat trace on the object surface can be described by the heat equation in a Cartesian coordinate system:

$$\frac{\partial u}{\partial t}=\alpha \left(\frac{\partial^{2}u}{\partial x^{2}}+\frac{\partial^{2}u}{\partial y^{2}}+\frac{\partial^{2}u}{\partial z^{2}}\right),$$ (4)

where (*x*, *y*, *z*) and *t* denotes the spatial variables and time variable of each point. In our case the object is assumed isotropic and homogeneous, thus the thermal diffusivity of the medium *α* will be constant. This equation indicates that the first-order derivative of temperature *U* related to time variable $\frac{\partial u}{\partial t}$ exhibits a linear relationship to the second-order derivative related to spatial variables $\frac{\partial^{2}u}{\partial x^{2}}+\frac{\partial^{2}u}{\partial y^{2}}+\frac{\partial^{2}u}{\partial z^{2}}$ . Also the divergence of a 3D temperature field can be solved by

$$Divergence=\frac{\partial^{2}U}{\partial x^{2}}+\frac{\partial^{2}U}{\partial y^{2}}+\frac{\partial^{2}U}{\partial z^{2}}.$$ (5)

We do not have to perform heat conduction simulation, but only to find the divergence of each point in the temperature field. Then the point with a lower divergence (faster cooling) has a greater probability to be a finger-object contact point. Hence, the numerical solution of these three second-order partial derivative terms $\frac{\partial^{2}u}{\partial x^{2}}$ , $\frac{\partial^{2}u}{\partial y^{2}}$ and $\frac{\partial^{2}u}{\partial z^{2}}$ at each point in the candidate region need to be calculated.

Unfortunately, for a non-grid or meshless structure such as point cloud, the common second-order central difference formula

$$\frac{\partial^{2}u}{\partial x^{2}}\approx \frac{U\left(x_{0}+\Delta x,y_{0},z_{0}\right)-2U\left(x_{0},y_{0},z_{0}\right)+U\left(x_{0}-\Delta x,y_{0},z_{0}\right)}{\Delta x^{2}}$$ (6)

is not available. Because in the point cloud, which cannot be ensured, the temperature at both of the two points (*x*_0_ + Δ*x*, *y*_0_, *z*_0_) and (*x*_0_ − Δ*x*, *y*_0_, *z*_0_) can be observed by the cameras at the same time. To solve this problem, we propose a fast meshless finite difference method, in which a temperature difference field needs to be firstly calculated for each point in the candidate region. Then a system of differential equations based on Taylor expansion will be solved using an elimination method for calculating the divergence.

### 6.1 Temperature difference along each axis

Given a point *P*_0_ in the candidate region, a further neighborhood search is performed for *P*_0_ to obtain its neighbor point set. Then a vector set of temperature difference $\vec{\Delta U}$ between each neighbor points and *P*_0_ is calculated. In which, $\vec{\Delta U_{i}}$ is a vector whose direction is the same as $\vec{P_{0}P_{i}}$ and its norm equals the temperature difference between the points *P*_*i*_ and *P*_0_. Then based on $\vec{\Delta U}$ , the components of temperature difference along the X-axis, Y-axis and Y-axis directions Δ*U* ^*x*^, Δ*U* ^*y*^ and Δ*U* ^*z*^ can be calculated using

$$\Delta U_{i}^{k}=\|\vec{P_{i}^{x}}\|\frac{\left\|\vec{\Delta U_{i}}\right\|}{\left\|\vec{P_{0}P_{i}}\right\|};\;i=\left[1,Num\right];\;i\in N;\;k\in \left\{x,y,z\right\},$$ (7)

where $\vec{P_{i}^{x}}$ denotes the X-component of $\vec{P_{0}P_{i}}$ along the X-axis and *Num* denotes the number of points in the neighborhood of *P*_0_.

### 6.2 Solving second-order derivative

It is well known that the Taylor series is fundamental for solving partial differential equations. In our case, if Δ*x* ≠ 0, Δ*y* = 0 and Δ*z* = 0, the second-order Taylor expansion of *U*(*p*) for a 3D point *p*_0_ (*x*_0_, *y*_0_, *z*_0_) and one of its neighbor point *p*_*i*_ (*x*_0_ + Δ*x*, *y*_0_ + Δ*y*, *z*_0_ + Δ*z*) is

$$U\left(x_{0}+\Delta x,y_{0},z_{0}\right)=U\left(x_{0},y_{0},z_{0}\right)+\Delta xU_{x}^{\prime }+\frac{\Delta x^{2}}{2}U_{xx}^{\prime \prime }+E_{2},$$ (8)

where *E*_2_ denotes a second-order error term. In our case, it is considered to be approximately equal to zero. We use an elimination method to solve this system of equations. Thus a coefficient vector *A* will be introduced and each Taylor expansion for *p*_0_ and each neighbor point *p*_*i*_ is multiplied by the coefficient *a*_*i*_ ∈ *A* and then summed to obtain

$$\begin{array}{cc} A\cdot {\left(U_{0}+\Delta U^{x}\right)}^{T} & \approx A\cdot U_{0}^{T}+C_{1}U_{x}^{\prime }+C_{2}U_{xx}^{\prime \prime }\\ C_{1} & =A\cdot \Delta X^{T}\\ C_{2} & =\frac{1}{2}A\cdot {\left(\Delta X◦\Delta X\right)}^{T}, \end{array}$$ (9)

where Δ*U* ^*x*^ denotes a vector consisting the x-components of temperature difference between *p*_0_ and *p*_*i*_ that was obtained in the previous section. Δ*X* is a vector consisting of the X-components of distances between *p*_0_ to *p*_*i*_. *U*_0_ denotes a vector consisting of the temperature at the point *P*_0_, in which all the elements are equal. We need to find the coefficient vector *A* that meets the conditions *C*_1_ = 0 and *C*_2_ ≠ 0. Then the second-order partial derivative of *U* at the point *p*_0_ with respect to *x* can be solved using

$$\frac{\partial^{2}U}{\partial x^{2}}\approx \frac{2A\cdot \Delta {U^{x}}^{T}}{A\cdot {\left(\Delta X◦\Delta X\right)}^{T}}.$$ (10)

In Supplementary Appendix SC, an algorithm for solving this system of differential equations will be described in detail. Similarly, $\frac{\partial^{2}U}{\partial y^{2}}$ and $\frac{\partial^{2}U}{\partial z^{2}}$ can be solved for and the divergence can be calculated using Eq. 5.

### 6.3 Likelihood calculation

A likelihood function related to the divergence field is defined as

$$L\left(x\right)=\int L\left(D\left(x\right)\right)dx,$$ (11)

where *x* denotes the spatial variables (point position) in the candidate region. The function *D*(*x*) describes the divergence distribution, i.e., Eq. 5. *L*(*D*(*x*)) refers to the likelihood distribution negatively relative to the divergence distribution, as shown in Figure 3B. Finally, by using the Eqs 1, 2, the real finger-object contact points will be calculated.

## 7 Robot motion trajectory calculation (module 4)

Furthermore, the normal vector of each contact point will be calculated using the 3D-data to allow that the robot moves always perpendicular to the object surface. However, it is obvious that the resolution of the trajectory obtained by this method is limited by the width of the finger. Hence, in response to this, we have to perform linear interpolation twice, the first time in 3D spatial space to achieve a smooth path (positions and orientations) for the end effector and the second time in robot joints space ensure limited angular velocity for the robot joint motion.

## 8 Experiments

### 8.1 Experiment for divergence solution

**FIGURE 4:**
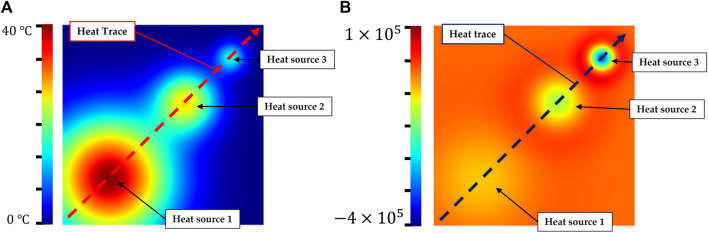
Simulated test data for the experiment to evaluate the accuracy of divergence solution. **(A)**: simulated thermal point cloud with a trajectory including three Gaussian point heat sources **(B)**: the ground truth of divergence solved by the second-order central difference formula.

In this section we will present an experiment to evaluate the accuracy of the divergence solution. In order to avoid the influence of sensor-specific noise on evaluation results, we built a uniform regular point cloud that can be gridded, as shown in Figure 4A. This simulated data was a board with a spatial resolution of 0.5 mm as well as its length and width are 0.1 m. A temperature field was initialized with a heat trace caused by three different Gaussian point heat sources. The parameters of these heat sources are shown in Table 1.

**TABLE 1:** The parameters for three Gaussian point heat sources.

	Heat source 1 (bottom left)	Heat source 2 (middle)	Heat source 3 (top right)
**Amplitude**	40°C	30°C	20°C
**Standard deviation**	0.01 m	0.005 m	0.001 m

Heat source 3 has a lower amplitude and standard deviation compared to heat sources 1 and 2. This indicates that heat source 3 is a new heat source, but it has a lower temperature than others. In contrast, heat source 1 has the highest temperature but it is the oldest heat source (Gaussian function with a lowest standard deviation). Hence, these three heat sources (*hs*_1_, *hs*_2_ and *hs*_3_) at time *t*_1_, *t*_2_ and *t*_3_ have temperature that consistent with 1 > 2 > 3, and their divergence were consistent with 1 < 2 < 3, as shown in the Figure 4. The experiment was set up in this way because the residual temperature of the finger on the object depends on the contact area between the finger and the object as well as the duration of contact. In other words, a new contact point is not certainly hotter than an old one.

By using the finite difference method (Eq. 6), the ideal divergence was calculated as ground truth from the uniform grid data, as shown in Figure 4B. The point cloud was then randomly downsampled into meshless data. Finally, our method was utilized to solve partial derivative solution in the sampled meshfree point cloud.

**FIGURE 5:**
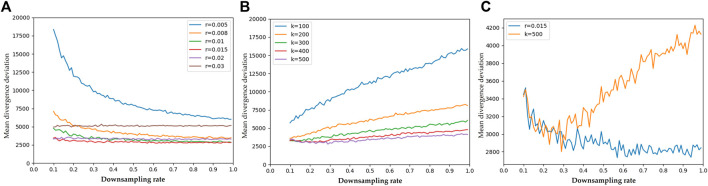
The results of the experiments to evaluate the accuracy of divergence solution. **(A)**: mean deviation of divergence using RNN neighbor search with various search radius *r* = [0.005 m, 0.03 m] and downsampling rate *r* = [0.1, 0.99] **(B)**: mean deviation of divergence using KNN neighbor search with various number of neighbors *k* = [100, 500] and downsampling rate *r* = [0.1, 0.99] **(C)**: comparison between the best results using RNN (*r* = 0.015 m) and KNN (*k* = 500).

In order to solve the divergence, a further neighborhood search should be performed for each point in the candidate region. In this experiment, two common neighborhood search methods (k-nearest neighbor (KNN) and radius nearest neighbor (RNN)) were evaluated. Figure 5 shows the results (mean divergence deviation for each point in sampled point clouds) by using RNN with various search radius *r* = (0.005 m, 0.03 m) and using KNN with various number of neighbors *k* = (100, 500). Meanwhile, the uniform regular point cloud was downsampled with various downsampling rate *β* = (0.1, 0.99). This downsampling rate is defined as

$$\beta =\frac{N_{\mathrm{sampled}}}{N_{\mathrm{original}}},$$ (12)

where *N*_*sampled*_ and *N*_*original*_ denote the number of points in the sampled and original point cloud.

**FIGURE 6:**
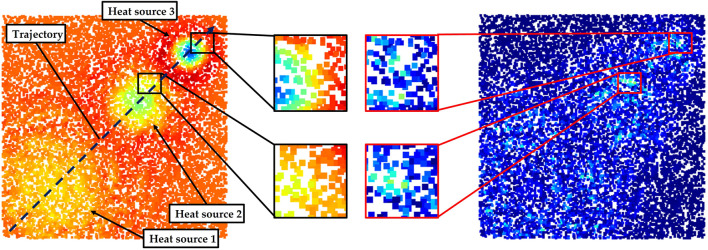
Visualization of a calculated divergence distribution (left) and the corresponding error distribution (right) using our method, where brighter points in the error distribution indicate greater errors.

The results of RNN show that the radius of 0.015 m has the lowest mean error. It is also robust towards a variety of downsampling rates. The radius of 0.005 m has the worst results and there is a clear tendency for the results to be worse as the downsampling rate *β* is decreased. This is because the random downsampling not only leads to a reduction of the spatial resolution of point cloud, it also causes some random defects in the point cloud. These defects result in a non-uniform distribution of samples in the eight quadrants of neighborhoods for solving the divergence at a point. The non-uniformity significantly affects the accuracy of the solution for divergence. As shown in Figure 6, the left point cloud shows the predicted divergence and the absolute deviation for each point is presented by the right cloud, with the brighter points having a greater error. Obviously, the error is larger in areas where the neighbor samples are unevenly distributed and where the absolute value of the divergence is great (there is a strong positive or negative heat transfer). A straightforward solution to this problem is to dilute the non-uniformity with a large search radius. However, unfortunately a large search radius also dilutes the fine-grained information. Therefore, with a radius of 0.03 m, the error is consistently large, although there is robustness towards different downsampling rates, as shown in Figure 5A. This is also reflected in the results of KNN (as shown in Figure 5B). With a fixed number of neighbors, the perceptual field of the neighborhoods grow up as the sampling rate increases, leading to more errors. Figure 5C shows a comparison of the best results by RNN and KNN respectively. It is clear that RNN has better robustness than KNN, which is in line with our expectations. In the point clouds captured by a real 3D sensor, some defects will inevitably appear because there are always some object points that cannot be 3D reconstructed. The neighborhood determined by KNN cannot even ensure that the target point is located in the geometric center of the neighborhood. Therefore, in our case it is an optimal choice to adopt a suitable search radius, provided the spatial resolution of the 3D sensor is already known.

**FIGURE 7:**
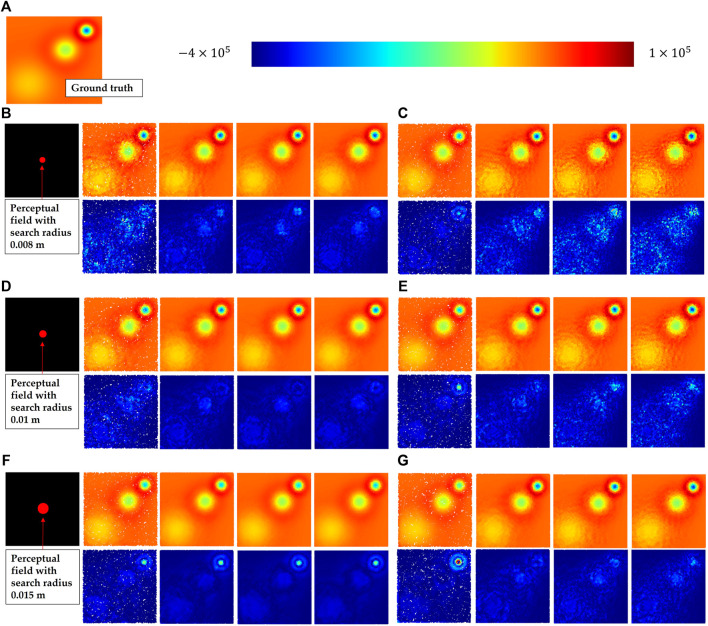
These figures show the results of the experiment to evaluate the accuracy of divergence solution. **(A)** Shows the ground truth of the divergence distribution; then, the results (top) and errors (bottom) using RNN with search radius (top to bottom: 0.008 m **(B)**, 0.01 m **(D)** and 0.015 m **(F)**) are presented; finally, the results using KNN with number of neighbors (top to bottom: 200 **(C)**, 300 **(E)** and 500 **(G)**) are presented. Each subfigure also shows the results with various same downsampling rates (left to right: 0.1, 0.4, 0.7, 0.9).

Figure 7 shows some visualization of this experiment. Figure 7A presents the ground truth of the divergence field. Then, Figures 7B, D, F exhibit the results by RNN with search radius of 0.008 m, 0.01 m and 0.015 m respectively, where the top-left graph shows the perceptual field of the search radius. The following presents in turn the results (top) and errors (bottom) for sampled point clouds with various downsampling rates of 0.1,0.4,0.7 and 0.9. Figures 7C, E, G show the results and errors of KNN with number of neighbors of 200, 300 and 500 towards the identical downsampling rates respectively. In the error plots, brighter points denote higher errors.

The results of RNN show that there is the lowest mean error at the search radius 0.015 m. However, compared to the result of the radius 0.01 m, it has a noticeably larger error in the nearby region of heat source 3 *hs*_3_. It confirms what was aforementioned, that too large a search radius will dilute the fine-grained information and lead to deviation. In fact, we always focus more on these high frequency areas in practice, because in our case the points with the lowest divergence require more attention. Therefore, the principle for setting the search radius should be carried out by choosing the smallest radius while ensuring a uniform distribution of samples in the neighborhood. Compared to RNN, KNN performs weakly in both mean error and fine-grained error.

### 8.2 Experiments for linear finger trace estimation

**FIGURE 8:**
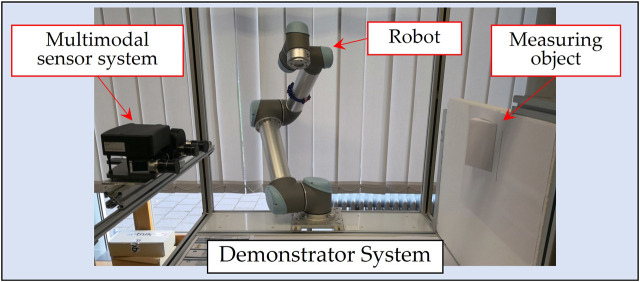
Experiment environment and our demonstrator system.

**FIGURE 9:**
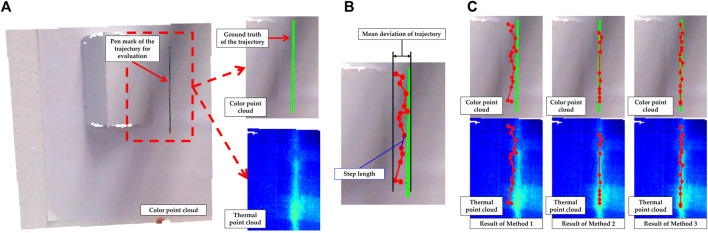
**(A)** Shows the straight line pen mark on the object surface (left), the ground truth of finger movement (top left) and the correspondent thermal point cloud **(B)** shows the evaluation criteria (Mean deviation of the trajectory and step length between each two adjacent contact points of the trajectory) of the experiment to evaluate the estimation for a straight line finger trajectory **(C)** shows the estimated contact points of a trajectory using method 1 (left), method 2 (middle) and method 3 (right), which are presented with color point cloud (top) and thermal point cloud (bottom). (Method 1 (left): anchor points were defined directly as contact points. This means that this method is not based on temperature analysis. Method 2 (middle): contact points were estimated using likelihoods that were positively correlated with temperature in the neighborhoods of the anchor points. In other words, in this method the temperature is analyzed as a static feature similar to color. In method 3 (right), likelihoods negatively dependent on the divergence were used for prediction (our method)).

In the subsequent experiments, the performance of our finger recognition method will be validated in a real environment. As shown in Figure 8, the measuring object is placed roughly 1 m in front of the sensors and the robot is situated between them. A 15 cm long straight line mark was drawn on the object surface with a pen. In the multimodal point cloud, the points belonging to this line were found based on color and further in fitting a 3D straight line as ground truth of a finger trajectory, as shown in Figure 9A. Then the finger drew a heat trace along this line, which was repeated 30 times. Finally, 15 contact points were estimated on each heat trace by using three different methods, as shown in Figure 9C. Method 1 (left): anchor points were defined directly as contact points. This means that this method is not based on temperature analysis. Method 2 (middle): contact points were estimated using likelihoods that were positively correlated with temperature in the neighborhoods of the anchor points. In other words, in this method the temperature is analyzed as a static feature similar to color. In method 3 (right), likelihoods negatively dependent on the divergence were used for prediction (our method).

The first row in Table 2 shows the mean deviation (as shown in Figure 9B) of the results by using these three methods on the 30 heat traces relative to ground truth. It is obvious that method 2 and method 3 have significantly lower deviation than method 1. It confirms that the application of multimodal data processing provides significant enhancement for this task. In Figure 9C, by method 2, it is surprising that the number of contact points which can be observed is less than 15, due to the overlap of multiple points. In fact, when this heat trace was created, the finger pressure on the object surface was not homogeneous, leading to excessive temperatures in some areas than others. Around these areas, the temperature-related likelihood (method 2) provides incorrect information, guiding the contact points to a deviated position (hottest position). In this respect, we further calculated the standard deviation of the step length between each two adjacent contact points of a trajectory (as shown in Figure 9B) using

$$std=\sqrt{\frac{1}{N}\overset{N}{\underset{i=1}{\sum }}|s_{i}-\bar{s}{|}^{2}},$$ (13)

where $\bar{s}$ denotes the mean step length of a trajectory and *N* denotes the contact point number of a trajectory. The results are shown in the second row of Table 2. It exhibits that the contact points in trajectories obtained by method 2 always have non-uniform step length. In the case where the finger trajectory is no longer a straight line but an arbitrary curve, it results in the robot trajectory being interpolated not smoothly and imprecisely. In the next experiment, this hypothesis will be confirmed.

**TABLE 2:** Mean deviation and step length standard deviation of the contact point estimation using three methods.

	Method 1 (mm)	Method 2 (mm)	Method 3 (mm)
**Mean deviation**	9.046	2.842	2.185
**Standard deviation of step length**	3.989	7.943	3.506

### 8.3 Experiments for arbitrary finger trace estimation

**FIGURE 10:**
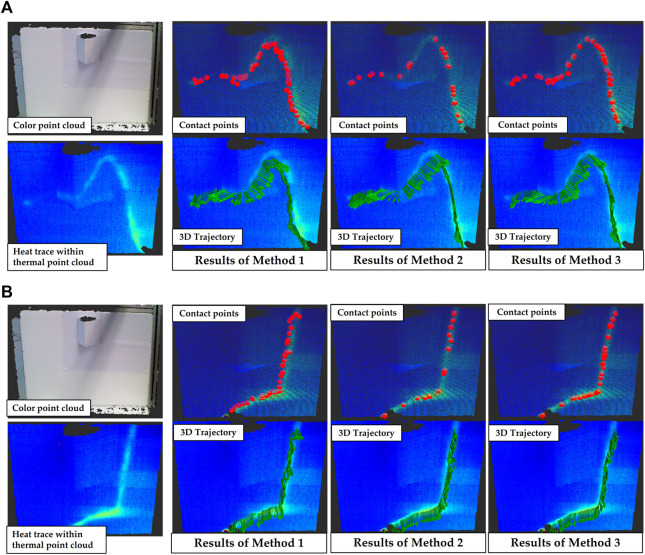
Robot motion trajectory predictions for two arbitrary finger movements are shown in figures **(A,B)**. In each subfigure, the first column shows a color object point cloud (top) and a residual heat trace presented within a thermal point cloud (bottom); the following columns show the predicted contact points (top) and interpolated 3D trajectories (bottom) obtained using various three methods. (left to right: Method 1: anchor points were defined directly as contact points. This means that this method is not based on temperature analysis. Method 2: contact points were estimated using likelihoods that were positively correlated with temperature in the neighborhoods of the anchor points. In other words, in this method the temperature is analyzed as a static feature similar to color. In method 3, likelihoods negatively dependent on the divergence were used for prediction (our method)).

As shown in Figure 10, in this experiment two different arbitrary finger movements and the predicted robot motion trajectories are presented. In each subfigure, the image on the top left shows the target object, in the bottom left image the heat traces left on the object surface are displayed by a thermal point cloud. The second column of plots shows the predicted contact points by method 1 (top) and the robot motion trajectory obtained by interpolation based on these contact points (bottom). The third and last columns show respectively the results using method 2 and method 3.

It can be observed that the prediction of the trajectory calculated by method 1 are not accurate or smooth. Temperature-based (method 2) contact point prediction has improved in terms of accuracy (all of the contact points land within the hot areas). However, as mentioned previously, since the residual temperature depends on the touch area and touch duration between finger and object, the temperature of a new contact point is not definitely higher than an old one. Therefore, the temperature-based contact points will be clustered with multiple overlaps in high temperature regions, resulting in distorted and non-smooth interpolated trajectories. In contrast, the trajectory obtained by method 3 has significant advantages in terms of precision and smoothness.

## 9 Discussion and conclusion

This work proposed a multimodal vision-based robot teaching approach. By using RandLANet, Hand/object semantic segmentation is performed on multimodal 3D image data containing temperature, color and geometric features. Then a dynamic analysis for meshless 3D temperature field is achieved by our elimination method. Furthermore, the hand/object contact point is precisely estimated based on Bayesian theory. The experimental results show that based on our method, the multimodal information is sufficiently extracted, and the resulting robot motion trajectory has good accuracy (mean deviation: 2.185 mm) and smoothness.

We consider that for physical quantities such as temperature, for which the derivative related to time and spatial variables has a constant relationship, we should explore more deeply the useful information hidden behind them, rather than handling them as static features in the same way as color. This is a remarkable difference between multi-modal image processing and multi-channel or multi-spectral image processing.

In our method, semantic segmentation is achieved using a deep neural network technique and analysis of the temperature field is realized based on a traditional method (heat transfer equation). We believe that it is worth exploring to choose the occasion for neural network technology rationally when it is so widely applied nowadays. For example, when a validated physical model is already existent for temperature field analysis, traditional methods should be chosen. This will improve the interpretability of the entire system and reduce the strong dependence on datasets in similar deep neural networks.

The experiments proved that our schema works. It also raises a common problem for point cloud processing that the adjust of some parameters such as the search radius for neighborhood, is still based on experience. Also, the search radius in the RandLANet is actually a hyper-parameter that needs to be adjusted artificially. In the future, if an adaptively adjustable parameter mechanism can be devised, the technical barrier for robot teaching will be lowered even further.

Moreover, this article represents only the first step of our Long-Term plan. Our future goal is to develop a self-heating pen that is much cheaper than Wandelbot’s TracePen, yet provides significantly higher accuracy than the finger-based method described in this article. Currently, a significant cause of trajectory errors (mean deviation: 2.185 mm) is due to the width of the finger, which is typically about 1 cm. A self-heating pen with a very fine nib can significantly improve accuracy. Additionally, a pen with adjustable temperature can help the system adapt to the effects of varying ambient temperatures. We are planning to increase the accuracy of this system to meet the low accuracy requirements of some industrial manufacturing scenarios. In such cases, implementing non-contact methods may be difficult to achieve.

## Data Availability

The raw data supporting the conclusion of this article will be made available by the authors, without undue reservation.
