# Supplementary material for: Interactive robot teaching based on finger trajectory using multimodal RGB-D-T-data
Source: Front Robot AI. 2023 Mar 16;10:1120357. doi: 10.3389/frobt.2023.1120357 (PMC10060539; doi:10.3389/frobt.2023.1120357)
Supplement: Supplementary file 1 [file Table1.pdf]

Supplementary Material

APPENDIX A

Given a hand point cloud HPC and an object point cloud OPC. Finding a nearest point in HPC for each point in OPC gives a set of Euclidean distances D:

$$D = \left\{ d | d = \min_{j} \| h_j - o_i \| \right\}$$
$$i = [1, N_{OPC}];\ j = [1, N_{HPC}];\ i, j \in \mathbb{N}\ , \quad \text{(S1)}$$

where N_{HPC} andN_{OPC} denote the number of points in HPC and OPC. Two distance thresholds d_1 and d_2 are introduced to extract two point sets P_1 and P_2 from OPC:

$$P_1 = \{p | p = o_i, d_i < d_1\}; P_2 = \{p | p = o_i, d_i < d_2\}$$
$$d_i \in D\ . \quad \text{(S2)}$$

A difference point set $P_{diff} = P_2 \setminus P_1$ is then obtained, which is an annular point cloud, as shown in figure **??**. We define the centroid of P_{diff} as the anchor point p_{anchor}, i.e.

$$p_{anchor} = \frac{1}{Num} \sum_{i=1}^{Num} p_i;\ p_i \in P_{diff}, \quad \text{(S3)}$$

where Num denotes the size of P_{diff}. The threshold d_1 should be set to slightly greater than the width of the finger. The setting of the threshold d_2 is dependent on the local density of the point cloud ρ and the threshold d_1:

$$d_2 = d_1 + k\rho, \quad \text{(S4)}$$

where k denotes the width of the annular point cloud. It influences whether samples can be uniformly obtained in each direction to estimate the anchor point.

APPENDIX B

Given two object point clouds OPC_{i-1} and OPC_i at time t_{i-1} and t_i as well as the anchor point p^{i-1}_{anchor}. Finding neighborhood in OPC_{i-1} and OPC_i for p^{i-1}_{anchor} gives two neighborhood point clouds N_{i-1} and N_i:

$$N = \{n | n = o_j, o_j < r\}\ ;\ j = [1, N_{OPC}];\ j \in \mathbb{N}\ , \quad \text{(S5)}$$

where r denotes a radius for neighborhood searching. Then finding a nearest point in N_{i-1} for each point in N_i gives a set of Euclidean distances D:

$$D = \left\{ d | d = \min_{j} \| n^{i-1}_j - n^i_k \| \right\}$$
$$j = [1, Num^{i-1}];\ k = [1, Num^i];\ i, j \in \mathbb{N}\ , \quad \text{(S6)}$$

where Num^{i-1} and Num^{i} denote the size of N_{i-1} and N_i. Then we need to determine the difference set of N_{i-1} and N_i. However, these two neighborhood point clouds are captured at different time, thus this difference set is not obtainable by using the set operators. Therefore, a threshold r_c was introduced to define the candidate region point cloud C_{i-1} as

$$C_{i-1} = \left\{c|c = n_k^i, d_k > r_c\right\}; \; r_c = \alpha\rho; \; d_k \in D \; , \tag{S7}$$

where r_c denote the tolerance of the overlap ratio of each nearest point pair of N_{i-1} and N_i. If they have a distance d_k less than r_c, these two points are considered to be approximately coincident. Therefore, r_c depends on a gain factor α and the local density ρ in the point cloud, where α should be set to $1 < \alpha < 2$ to ensure that there are no outliers or defects in the candidate region point cloud C_{i-1}.

APPENDIX C

Given the system of second-order differential equations:

$$\begin{aligned} A \cdot (U_0 + \Delta U^x)^T &\approx A \cdot U_0^T + C_1 U_x^{'} + C_2 U_{xx}^{''} \\ C_1 &= A \cdot \Delta X^T \\ C_2 &= \frac{1}{2} A \cdot (\Delta X \circ \Delta X)^T \; . \end{aligned} \tag{S8}$$

In order to get the solution of $U_{xx}^{''}$, we need to eliminate the terms $U_x^{'}$ from the equations, i.e. $C_1 = 0$. Obviously, there are an infinite number of coefficient vectors, which are parallel to the hyperplane with ΔX as the normal vector, meets this condition. Firstly, we split ΔX into three vectors ΔX_0, ΔX_1 and ΔX_2. ΔX_0 consists of all zero elements in ΔX, ΔX_1 consists of all positive elements and the remaining negative elements compose ΔX_2. If ΔX_1 and ΔX_2 are not empty sets $\emptyset$, according to geometric definition of the dot product of two vectors:

$$A \cdot B = \|A\| \|B\| \cos\theta \tag{S9}$$

let $\theta = 0$, then the coefficient vector A_1 and A_2 for ΔX_1 and ΔX_2 can be calculated using

$$A_1 = \frac{|\Delta X_1|}{\|\Delta X_1\|^2}; \; A_2 = \frac{|\Delta X_2|}{\|\Delta X_2\|^2} \; , \tag{S10}$$

where $|\Delta X_1|$ and $|\Delta X_2|$ denote two vectors consisting of the absolute values of the elements in ΔX_1 and ΔX_2. Thus A_1 and A_2 meet the following conditions:

$$A_1 \cdot \Delta X_1^T = 1; \; A_2 \cdot \Delta X_2^T = -1 \; , \tag{S11}$$

The coefficient vector A_0 for ΔX_0 can be defined as a vector with only zeros. Therefore, the vector A composed of A_0, A_1 and A_2 meets the condition $C_1 = 0$. On the other hand, it is also easy to prove $C_2 \neq 0$. By using this algorithm, in this system of equations, the first-order terms are eliminated and the second-order terms are retained. For solving the second-order partial derivative with respect to x, the above equations can be organized as

$$\frac{\partial^2 U}{\partial x^2} \approx \frac{2A \cdot |\Delta U^x|^T}{A \cdot (\Delta X \circ \Delta X)^T} \; . \tag{S12}$$
